# On the statistical significance of communities from weighted graphs

**DOI:** 10.1038/s41598-021-99175-2

**Published:** 2021-10-13

**Authors:** Zengyou He, Wenfang Chen, Xiaoqi Wei, Yan Liu

**Affiliations:** 1 School of Software, Dalian University of Technology, Dalian, 116024 China; 2 Key Laboratory for Ubiquitous Network and Service Software of Liaoning Province, Dalian, 116024 China

**Keywords:** Computer science, Statistics

## Abstract

Community detection is a fundamental procedure in the analysis of network data. Despite decades of research, there is still no consensus on the definition of a community. To analytically test the realness of a candidate community in weighted networks, we present a general formulation from a significance testing perspective. In this new formulation, the edge-weight is modeled as a censored observation due to the noisy characteristics of real networks. In particular, the edge-weights of missing links are incorporated as well, which are specified to be zeros based on the assumption that they are truncated or unobserved. Thereafter, the community significance assessment issue is formulated as a two-sample test problem on censored data. More precisely, the Logrank test is employed to conduct the significance testing on two sets of augmented edge-weights: internal weight set and external weight set. The presented approach is evaluated on both weighted networks and un-weighted networks. The experimental results show that our method can outperform prior widely used evaluation metrics on the task of individual community validation.

## Introduction

Community detection is a fundamental issue in network data analysis. It aims at dividing nodes in a network into different groups called communities. It is expected that there should be more edges within each community and few edges across different communities. The community detection procedure has been widely used in many fields such as social science, biology, medicine, and chemistry^1^.

During the past decades, numerous community detection algorithms have been developed from different perspectives^1–4^. Despite these developments, the issue of deciding whether a derived community is real or not is far from being resolved. Such a community validation issue fits naturally into a framework of hypothesis testing, in which the null hypothesis is that the target community is not real.

In fact, many metrics such as modularity and conductance have been proposed for assessing the goodness of a potential community^5^. However, most of these metrics are not developed based on a rigorous significance testing procedure^6^. Theoretically, the realness of a community should be an analytical problem relative to some particular definitions of communities. Towards this direction, several research efforts have been conducted to analytically assess the realness of one candidate community, such as OSLOM^7,8^ , ESSC^9^, DSC^10^, CCME^11^, and FOCS^12^. Among these methods, only OSLOM and CCME focus on validating a community in weighted networks. Unfortunately, in both OSLOM and CCME, the statistical significance of a target community is assessed through the probability of association between each node and the community^13^. One method that can directly test the realness of a community in edge-weighted graphs is still not available.

We formulate the community significance assessment problem in edge-weighted networks as a non-parametric two-sample test issue on censored data. In this paper, the edge-weights are assumed to be non-negative and continuous. The network structure and edge-weights may contain substantial measurement errors during the network inference process^14^. Based on this observation, we model each edge-weight as a censored observation in survival analysis^15^. If there is no edge between two nodes, the corresponding edge-weight is 0, which is either unobserved or truncated. Consequently, we construct two groups of edge-weights: one group is composed of edge-weights within the community and another group is composed of edge-weights between nodes in the community and remaining nodes outside the community. If the target community is not a real community, it is reasonable to expect that there is no difference between these two groups. Therefore, we can utilize a distribution-free two-sample test procedure in censored data analysis to assess the statistical significance of the candidate community. In this paper, we choose the popular Logrank test^16^ to fulfill this task.

One of the characteristics of the presented formulation is that the unreliability of edge-weights is fully incorporated into the model. Meanwhile, it provides a general framework for validating weighted communities from a new angle. In particular, for un-weighted networks, we can reveal some interesting connections between our formulation and the community evaluation metric in Ref.^17^. We evaluate our method on both weighted networks and un-weighted networks. Experiments demonstrate that our method is comparable with prior state-of-the-art metrics on individual community assessment.

The rest of this article is arranged as follows. In the next section, our formulation is described in detail. Thereafter, the experimental results are presented and some discussions are given in the last two sections.

## Method

### Notations

Given a weighted network $G = (V,E,W)$, where *V* is the node set, *E* is the edge set, and *W* is the set of positive weights. For a given subset of nodes *S* ($S \subseteq V$), its induced subgraph *G*[*S*] can be regarded as a candidate community. All edges incident on the nodes in *S* could be divided into two groups: the set of internal edges within *G*[*S*] that are incident on two nodes from *S* and the set of external edges of *G*[*S*] which are incident on one node from *S* and another node from $V \backslash S$.

### Formulation

**Figure 1:**
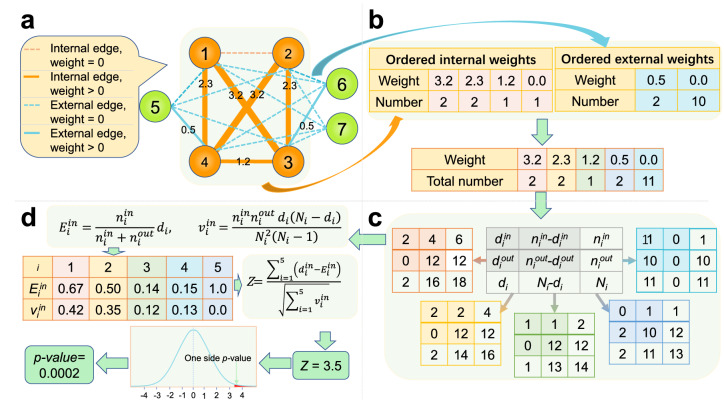
The main workflow of our method. (**a**) The network with augmented edge-weights for missing links. Each solid line represents a real edge and each dashed line denotes an augmented edge that is not included in *E*. In this network, there are totally 7 nodes and the candidate community is the sub-graph induced by the node set {1,2,3,4}. The size of each line is proportional to the corresponding edge-weight and the weights of augmented edges for missing links are zeros. (**b**) The ordered edge-weights. For the given candidate community in (**a**), we can construct two sets of edge-weights. One set is composed of the internal edge-weights and another set is composed of the external edge-weights. We can order these two sets to check if their empirical cumulative distribution functions are different. If the candidate community is not a real one, it can be expected that these two sets of weights have the same distribution. In Logrank test, these two sets are merged to generate a combined ordered edge-weight list. (**c**) Contingency tables for distinct weights. For each of the five distinct weights in (**b**), we can construct a contingency table. For instance, there are 2 edges whose weights are 3.2 and these two edges are internal edges, the first column of the leftmost table is (2, 0, 2). The second column of the leftmost table is (4, 12, 16) since there are 4 (and 12) internal (and external) edges whose weights are less than 3.2. (**d**) The final test statistic and its formula. Based on the contingency tables in (**c**), we can calculate the test statistic *Z* and the corresponding *p*-value to quantify the statistical significance of the candidate community. The Bonferroni correction is further applied to deliver an adjusted *p*-value, which is obtained by multiplying the original *p*-value with number of all possible communities of the same size.

To test if a given candidate community in the weighted network is a real one, we present a formulation based on the non-parametric two-sample test for censored data. The main workflow of our method is shown in Fig. 1, which will be elaborated below.

In the first step of our method, we regard each edge-weight as a censored observation. As shown in Fig. 1*a*, the set of edge-weights is augmented by including the weights of missing links. The edge-weights of these missing links are 0s since they are either truncated or unobserved. Then, we can construct two augmented sets of edge-weights: the set of internal edge-weights $$W_{S}^{in} = \{w_{1}^{in}, w_{2}^{in},\ldots, w_{\left( {\begin{array}{c}|S|\\ 2\end{array}}\right)}^{in}\}$$ and the set of external edge-weights $W_{S}^{out} = \{w_{1}^{out}, w_{2}^{out},\ldots, w_{|S|(|V|-|S|)}^{out}\}$.

Let $F_{in}(\cdot )$ and $F_{out}(\cdot )$ be complementary cumulative distribution functions for internal and external weights, respectively. Then, the community assessment issue can be modeled as a two-sample test problem, in which the hypotheses under consideration are:

$$H_{0}$$: $$F_{in}(w)=F_{out}(w)$$;

$H_{1}$: $F_{in}(w)>F_{out}(w)$, for at least one *w*, where $w\ge 0$ is the non-negative weight.

Note that a larger edge-weight indicates a stronger connection between two corresponding nodes and both $F_{in}(\cdot )$ and $F_{out}(\cdot )$ are complementary cumulative distribution functions. If $H_{0}$ is violated and $H_{1}$ holds, then there will be more internal edges with positive edge-weights and the internal edges are associated with larger weights. Hence, the proposed two-sample test is capable of quantifying the realness of a target community in a statistically sound manner.

To solve the above two-sample test issue, many effective methods can be utilized. Here we adopt the Logrank test due to its popularity in censored data analysis. As shown in Fig. 1b, $W_{S}^{in}$ and $W_{S}^{out}$ are first merged to generate a new set $W_{S}$ and then all edge-weights in $W_{S}$ are sorted in a non-increasing order. For each distinct edge-weight $w_{i}$ in $W_{S}$, we can construct a $2\times 2$ table as shown in Fig. 1c. In Fig. 1c, $d_{i}^{in}$, $d_{i}^{out}$ and $d_{i}$ denote the number of internal edges, the number of external edges and the number of edges in $W_{S}$ whose edge-weights are $w_{i}$. Meanwhile, $n_{i}^{in}$, $n_{i}^{out}$ and $n_{i}$ denote the number of internal edges, the number of external edges and the number of edges in $W_{S}$ whose edge-weights are not bigger than $w_{i}$.

Based on above notations, the test statistic *Z* of Logrank test is:

1 $$\begin{aligned} Z = \frac{\sum _{i=1}^{q}(d_{i}^{in}-E_{i}^{in})}{\sqrt{\sum _{i=1}^{q}v_{i}^{in}}}, \end{aligned}$$

where *q* is the number of distinct edge-weights in $W_{S}$, $N_i=n_{i}^{in}+n_{i}^{out}, E_i^{in} = \frac{n_{i}^{in}}{N_{i}}d_{i}$ and $v_i^{in} = \frac{n_{i}^{in} d_{i}(N_{i}-d_{i})n_{i}^{out}}{(N_{i})^2(N_{i}-1)}$. When $H_{0}$ is true, *Z* approximately follows a *N*(0, 1) distribution. In Eq. (1), each ($d_i^{in}-E_i^{in}$) can be regarded as the “observed minus expected” difference with respect to the number of internal edges of a specific weight. Thus, a real community should be associated with a large *Z* statistic. Based on this approximation, the corresponding *p*-value can be obtained to assess the statistical significance of *G*[*S*].

In regard to the combinatorial nature of community detection, the community significance assessment issue is actually a multiple hypothesis testing problem. Hence, we need to conduct a multiple testing correction by calculating an adjusted *p*-value for each candidate community. The most popular method for multiple testing correction is probably the Bonferroni correction approach, in which the original *p*-value is multiplied by the number of tested hypotheses to obtain an adjusted *p*-value. In our context, the number of tested hypotheses can be calculated as the number of possible communities of the same size. More precisely, for a given community *G*[*S*] of size |*S*|, its adjusted *p*-value is calculated as $$p_{adj}(G[S])=min\{1, p(G[S])\times \left( {\begin{array}{c}|V|\\ |S|\end{array}}\right) \}$$, where *p*(*G*[*S*]) is original *p*-value of *G*[*S*] and |*V*| is the number of nodes of the graph. In the experiment, the adjusted *p*-value is used instead of the original *p*-value in our method.

### Un-weighted special case

For un-weighted networks, the edge-weight is either 1 or 0, and thus $q=2$ in Eq. (1). For the weight $w_1 = 1$, we have: $$n_{1}^{in} = \left( {\begin{array}{c}|S|\\ 2\end{array}}\right) $$, $n_{1}^{out} = |S|(|V|-|S|)$, and thus $$E_1^{in} = \frac{n_{1}^{in}}{N_{1}}d_{1} = \frac{\left( {\begin{array}{c}|S|\\ 2\end{array}}\right) }{\left( {\begin{array}{c}|S|\\ 2\end{array}}\right) +|S|(|V|-|S|)}d_1$$, $$v_1^{in} = \frac{n_{1}^{in}n_{1}^{out}d_{1}(N_{1}-d_{1})}{(N_{1})^2(N_{1}-1)} = \frac{\left( {\begin{array}{c}|S|\\ 2\end{array}}\right) |S|(|V|-|S|)d_1\left[ \left( {\begin{array}{c}|S|\\ 2\end{array}}\right) +|S|(|V|-|S|)-d_1\right] }{\left[ \left( {\begin{array}{c}|S|\\ 2\end{array}}\right) +|S|(|V|-|S|)\right] ^{2}\left[ \left( {\begin{array}{c}|S|\\ 2\end{array}}\right) +|S|(|V|-|S|)-1\right] }$$. For the weight $w_2 = 0$, we have: $n_{2}^{in} = d_{2}^{in}$, $n_{2}^{out} = d_{2}^{out}$, $N_2 = d_2$, and thus $E_2^{in} = \frac{n_{2}^{in}}{N_{2}}d_{2} = d_{2}^{in}$, $v_2^{in} = 0$. Thus, Eq. (1) can be rewritten as:

2 $$\begin{aligned} \begin{aligned} Z&= \frac{(d_{1}^{in}-E_{1}^{in})+(d_{2}^{in}-E_{2}^{in})}{\sqrt{(v_{1}^{in}+v_{2}^{in})}} =\frac{(d_{1}^{in}-E_{1}^{in})}{\sqrt{v_{1}^{in}}} =\frac{(d_{1}^{in}-\frac{\left( {\begin{array}{c}|S|\\ 2\end{array}}\right) }{\left( {\begin{array}{c}|S|\\ 2\end{array}}\right) +|S|(|V|-|S|)}d_1)}{\sqrt{\frac{\left( {\begin{array}{c}|S|\\ 2\end{array}}\right) |S|(|V|-|S|)d_1\left[ \left( {\begin{array}{c}|S|\\ 2\end{array}}\right) +|S|(|V|-|S|)-d_1\right] }{\left[ \left( {\begin{array}{c}|S|\\ 2\end{array}}\right) +|S|(|V|-|S|)\right] ^{2}\left[ \left( {\begin{array}{c}|S|\\ 2\end{array}}\right) +|S|(|V|-|S|)-1\right] }}}\\&=\sqrt{\frac{\left( {\begin{array}{c}|S|\\ 2\end{array}}\right) (|S|(|V|-|S|))\left[ \left( {\begin{array}{c}|S|\\ 2\end{array}}\right) +|S|(|V|-|S|)-1\right] }{d_{1}\left[ \left( {\begin{array}{c}|S|\\ 2\end{array}}\right) +|S|(|V|-|S|)-d_{1}\right] }} \left[ \frac{d_{1}^{in}}{\left( {\begin{array}{c}|S|\\ 2\end{array}}\right) }-\frac{d_{1}^{out}}{|S|(|V|-|S|)}\right] \\&=\sqrt{\frac{\left( {\begin{array}{c}|S|\\ 2\end{array}}\right) \left[ \left( {\begin{array}{c}|S|\\ 2\end{array}}\right) +|S|(|V|-|S|)-1\right] }{(|S|(|V|-|S|))d_{1}\left[ \left( {\begin{array}{c}|S|\\ 2\end{array}}\right) +|S|(|V|-|S|)-d_{1}\right] }} |S|(|V|-|S|)\left[ \frac{d_{1}^{in}}{\left( {\begin{array}{c}|S|\\ 2\end{array}}\right) }-\frac{d_{1}^{out}}{|S|(|V|-|S|)}\right] . \end{aligned} \end{aligned}$$

In Ref.^17^, a new community evaluation metric has been presented, which can expressed as follows based on our notations:

3 $$\begin{aligned} \begin{aligned} Z'= |S|(|V|-|S|)\left[ \frac{d_{1}^{in}}{\frac{|S|^{2}}{2}}-\frac{d_{1}^{out}}{|S|(|V|-|S|)}\right] . \end{aligned} \end{aligned}$$

Thus, we can get the mathematical relation between *Z* (our test statistic for un-weighted networks) and $Z'$ (the community evaluation metric in Ref.^17^):

4 $$\begin{aligned} Z \approx Z' \sqrt{\frac{\left( {\begin{array}{c}|S|\\ 2\end{array}}\right) \left[ \left( {\begin{array}{c}|S|\\ 2\end{array}}\right) +|S|(|V|-|S|)-1\right] }{(|S|(|V|-|S|))d_{1}\left[ \left( {\begin{array}{c}|S|\\ 2\end{array}}\right) +|S|(|V|-|S|)-d_{1}\right] }}. \end{aligned}$$

As shown in Eq. (4), we can quantitatively establish the connection between the special case of our method for un-weighted networks and one popular metric in the literature.

## Results

**Figure 2:**
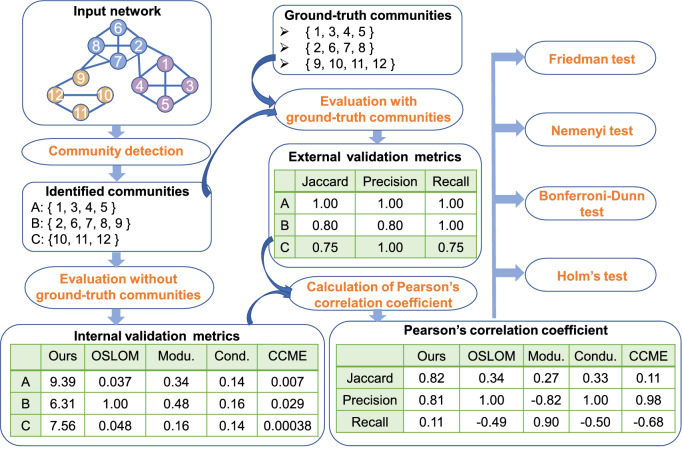
The main workflow of our experiments. Given a network with three ground-truth communities: {1, 3, 4, 5}, {2, 6, 7, 8} and {9, 10, 11, 12}, three communities are detected by the SLPAw algorithm: $A= \{1, 3, 4, 5\}$, $B= \{2, 6, 7, 8, 9\}$, $C=\{10, 11, 12\}$. Each identified community can be assessed using both the internal validation metric and the external validation metric. For example, the negative log value of our method and the precision for the identified community *A* is 9.39 and 1, respectively. Then, a vector for each metric on the set of identified communities is readily available. For instance, the validation index vector for our method and the Jaccard coefficient is (9.39, 6.31, 7.56) and (1, 0.8, 0.75), respectively. Consequently, the Pearson’s correlation coefficient is calculated between each pair of vectors: one from an internal validation metric and another one from an external validation metric. Based on the correlation coefficients, four statistical tests are further applied to check if our method is really better than other internal validation metrics on the task of individual community assessment.

To test whether the presented *p*-value is effective on community evaluation, we conduct a series of experiments according to the pipeline shown in Fig. 2. Firstly, existing community detection algorithms are employed to produce a set of identified communities on networks with ground-truth communities. Then, we use both internal validation metrics (e.g. our method, modularity) and external validation metrics (e.g. precision, recall) to quantitatively validate each identified community. Since external validation metrics are calculated based on the ground-truth information, which can be used as the “gold standard”. In other words, one internal validation metric is a good community validation index if it is highly correlated with each external validation metric on the assessment of identified communities. Based on this assumption, we calculate the Pearson’s correlation coefficient between two vectors (one is generated from an internal validation metric and another one is produced by an external validation metric), where each vector is composed of the validation index values on a set of identified communities. Finally, the Friedman test^18^ and three post-hoc tests: the Nemenyi test^19^, the Bonferroni–Dunn test^20^ and the Holm’s step-down test^21^ are employed to check if our method is significantly better than other popular internal validation metrics.

### Data sets

In our experiment, we use two groups of data sets. One group is composed of four weighted PPI (Protein–Protein Interaction) networks: Collins2007^22^, Gavin2006^23^, Krogan2006_core^24^, Krogan2006_extended^24^. There are three sets of ground-truth communities for these weighted PPI networks, where each set is collected from one of the following databases of protein complexes: CYC2008^25^, MIPS^26^ and SGD^27^. There are 408, 203 and 323 ground-truth communities in these three sets, respectively. Another group is composed of six real un-weighted networks: Karate^28^, Football^29^, Personal Facebook (Personal)^9^, Political blogs (PolBlogs)^30^, Books about US politics (PolBooks)^31^, and Railways^32^. The topological characteristics of six real un-weighted networks are provided in Supplementary Table S1.

**Figure 3:**
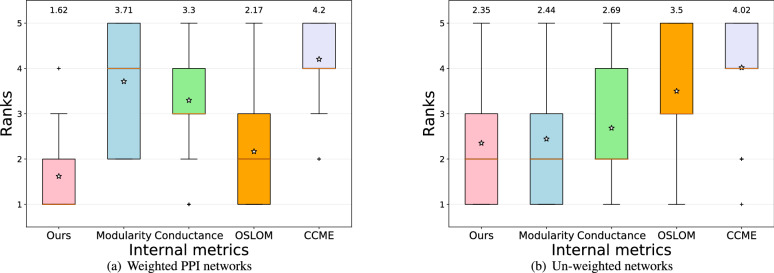
The rank distribution for each internal validation metric on (**a**) weighted PPI networks and (**b**) Un-weighted networks. Here the box plot is used to depict the distributions: minimum (lower line), lower quartile (lower edge of the box), median (center line), upper quartile (upper edge of the box) and maximum (upper line). Meanwhile, mean (star) and outlier (cross) are also plotted.

### Parameter setting

We choose three classical community detection methods: SLPAw^33^, Infomap^34^ and Louvain^35^ to detect communities. In our experiment, we run these three methods with their default parameter settings for weighted graphs.

### Experiment

We compare our method with four internal metrics: conductance^36^, modularity^37^, *p*-value in OSLOM^7^ and *p*-value in CCME^11^. The *p*-value of OSLOM is obtained with its default setting and the *p*-value of a community in CCME is the maximal *p*-value of all nodes within the community. Since Conductance, the *p*-values in our method, OSLOM and CCME are negatively correlated with Jaccard coefficient, Precision and Recall, we use the negative Pearson’s correlation coefficient for these three metrics in the performance comparison. Then, for the set of reported communities on each data set from each community detection algorithm, we can use the Pearson’s correlation coefficient with respect to each external validation metric to check which internal validation metric is better. More precisely, a better internal validation metric should have a larger correlation coefficient. The detailed results are recorded in the Supplementary Tables S2–S5. From these tables, we can obtain the rank distribution for each internal validation metric, where a larger correlation coefficient will be assigned to a smaller rank. The rank distributions on weighted PPI networks and un-weighted networks in terms of box plots are provided in Fig. 3a,b, respectively.

From Fig. 3, it can be observed that our method can achieve the smallest average rank among five internal validation metrics. To check if our method is really better than the other four internal validation metrics, we first apply the Friedman test to assess the null hypothesis that all methods have the same rank. The $\chi _{F}^{2}$ value in the Friedman test on weighted PPI networks and un-weighted networks is 200.5704 and 45.6889, respectively. This means that the performance gaps among different internal validation metrics are statistically significant when the significance level is specified to be 0.05. Then, we further employ the Nemenyi test, the Bonferroni-Dunn test and the Holm’s step-down test to compare our method with each competing internal validation metric in a pair-wise manner.

In Table 1, we record the rank difference between our method and each competing method on both weighted PPI networks and un-weighted networks. As shown in Table 1, the rank difference values between our method and Modularity, Conductance and CCME on weighted PPI networks are larger than the critical difference (CD) thresholds for both the Nemenyi test and the Bonferroni–Dunn test when the significance level is 0.05. This indicates that our method is significantly better than Conductance, Modularity and CCME under these two tests. On the un-weighted networks, our method is significantly better than OSLOM and CCME according to the Bonferroni–Dunn test and the Nemenyi test.

**Table 1:** The average rank difference RD(*a*, *b*) between two methods, where *a* denotes our method and *b* represents a competing internal validation metric.

	Weighted PPI networks	Un-weigthed networks
RD (Ours, modularity)	2.0926	0.0926
RD (Ours, conductance)	1.6759	0.3333
RD (Ours, OSLOM)	0.5463	1.1481
RD (Ours, CCME)	2.5833	1.6667
CD (Nemenyi)	0.8301	0.5870
CD (Bonferroni–Dunn)	0.7601	0.5375

In last two rows, the critical difference (CD) thresholds at the significance level $\alpha =0.05$ for the Nemenyi test and the Bonferroni–Dunn test are listed, respectively.

In Table 2, we list the *p*-values for the average rank difference between our method and each competing method based on the Holm’s step-down test. Besides, the adjusted significance level for each position after sorting the *p*-values in a non-decreasing order is provided as well. As shown in Table 2, all the *p*-values on PPI networks are smaller than the corresponding adjusted significance levels. This indicates the superiority of our method over other four metrics on weighted networks is also confirmed by the Holm’s step-down test. Similar to results in Table 1, we can claim that our method is significantly better than OSLOM and CCME on un-weighted networks based on the hypothesis testing results in Table 2.

**Table 2:** The *p*-value P(*a*, *b*) for the rank difference between two methods under the Holm’s step-down test on weighted PPI networks (the left column) and un-weighted networks (the right column), where *a* denotes our method and *b* represents a competing internal validation metric.

	Weighted PPI networks	Adjusted $\alpha$	Un-weigthed networks	
P (Ours, modularity)	0	0.0125	2.16E−08	P (Ours, CCME)
P (Ours, CCME)	0	0.0167	8.06E−05	P (Ours, OSLOM)
P (Ours, conductance)	3.33E−15	0.0250	0.1367	P (Ours, conductance)
P (Ours, OSLOM)	5.56E−03	0.0500	0.3805	P (Ours, modularity)

In the middle column, the adjusted significance level at $\alpha = 0.05$ for each position after sorting the *p*-values in a non-decreasing order is listed.

## Discussion

We have presented a general approach for assessing the statistical significance of a community from weighted networks. In this new formulation, the weights of missing links are set to be zeros and all edge-weights are treated as truncated observations. Based on this assumption, the community validation issue is modeled as a two-sample test problem on censored data. The presented formulation provides a general framework for community validation from a significance testing perspective. Based on this framework, we can either reveal the rationale underlying some existing community validation metrics or develop new community evaluation measures.

## Supplementary information

Supplementary Information.
