# Supplementary material for: On the statistical significance of communities from weighted graphs
Source: Sci Rep. 2021 Oct 13;11:20304. doi: 10.1038/s41598-021-99175-2 (PMC8514603; doi:10.1038/s41598-021-99175-2)
Supplement: Supplementary file 1 — Supplementary Information. [file 41598_2021_99175_MOESM1_ESM.pdf]

Supplementary document for "On the statistical significance of communities from weighted graphs"

Zengyou He
School of Software,
Dalian University of Technology, Dalian, China.
Key Laboratory for Ubiquitous Network and Service Software of
Liaoning Province, Dalian, China.
Email: zyhe@dlut.edu.cn

Wenfang Chen
School of Software,
Dalian University of Technology, Dalian, China.
Email: 2293508387@qq.com

Xiaoqi Wei
School of Software,
Dalian University of Technology, Dalian, China.
Email: 18398746998@163.com

Yan Liu
School of Software,
Dalian University of Technology, Dalian, China.
Email: 726078139@qq.com

Internal validation metrics

Definition

Four existing internal validation metrics: Modularity, Conductance and the p-value in OSLOM and the p-value in CCME are used in our experiment to validate each identified community.

- Modularity

  Modularity[1] is probably the most widely used community validation metric in the literature. The Modularity of an un-weighted community ξ is defined as:[1]

$$Modularity(\xi) = \frac{E_{in}}{|E|} + \left(\frac{E_{in} + E_{out}}{2|E|}\right)^2, \tag{1}$$

  where $|E|$ is the number of edges in the network and E_{in} (E_{out}) is the number of internal (external) edges of ξ. Accordingly, the Modularity of a weighted community C is defined as:

$$Modularity(C) = \frac{W_{in}}{|W|} + \left(\frac{W_{in} + W_{out}}{2|W|}\right)^2, \tag{2}$$

  where $|W|$ is the sum of edge-weights in the network and W_{in} (W_{out}) is the sum of internal (external) edge-weights of C.

- Conductance

  The Conductance[2] of an un-weighted community ξ is defined as:

$$Conductance(\xi) = \frac{E_{out}}{min(D_{\xi}, D - D_{\xi})}, \tag{3}$$

  where E_{out} is the number of external edges of the community, D_{ξ} is the total degree of the community and D is the total degree of the network. The Conductance of a weighted community C is defined as:

$$Conductance(C) = \frac{W_{out}}{min(S_C, S - S_C)}, \tag{4}$$

  where W_{out} is the sum of external edge-weights, S_C is the sum of edge-weights in C and S is the sum of edge-weights in the network.

- OSLOM

  Given a candidate community, its p-value is calculated as:

$$p = 1 - (1 - min_q\{\sum_{i=q}^{N-n_c} \binom{N-n_c}{i} r_q^i (1-r_q)^{N-n_c-i}\})^{N-n_c}, \tag{5}$$

  where n_c is the number of vertices in the candidate community, N is the total number of vertices in the network and r_q is the value of topological (and weight) information variable r with rank q of a node outside of the candidate community.

- CCME

  For a candidate community C, we use the maximum p-value of its internal nodes as the p-value of this community. The p-value of an internal node u is obtained as:

$$p(u) = 1 - \Phi\left(\frac{\sum_{v\in C} W_{uv} - \sum_{v\in C} \frac{s(u)s(v)}{\sum_{w\in N} s(w)}}{\sigma(u, C)}\right), \tag{6}$$

  where $\Phi(x)$ is the cumulative distribution function of variable x that satisfies the standard normal distribution,

$$\sigma(u, C) = \sqrt{\sum_{v\in C} \frac{s(u)s(v)}{\sum_{w\in N} s(w)} \frac{\frac{s(u)s(v)}{\sum_{w\in N} s(w)}}{min\left(1, \frac{d(u)d(v)}{\sum_{w\in N} d(w)}\right)} \left(1 - min\left(1, \frac{d(u)d(v)}{\sum_{w\in N} d(w)}\right) + \frac{\sum_{uv\in E}(W_{uv} - \frac{\frac{s(u)s(v)}{\sum_{w\in N} s(w)}}{min\left(1, \frac{d(u)d(v)}{\sum_{w\in N} d(w)}\right)})^2}{\sum_{uv\in E}(\frac{\frac{s(u)s(v)}{\sum_{w\in N} s(w)}}{min\left(1, \frac{d(u)d(v)}{\sum_{w\in N} d(w)}\right)})^2}\right)}, \tag{7}$$

  $s(x)$ ($d(x)$) is the strength (degree) of node x, W_{uv} is the edge-weight between node u and v, N is the node set and E is the edge set.

Complexity Analysis

For a given weighted network and a candidate community, the time complexity for calculating these compared internal metrics are listed as follows:

- The time complexity for calculating modularity and conductance are the same: $O(T)$, where T is the number of non-zero edge-weights associated with all internal nodes. Suppose the sum of all edge-weights is known in advance as a constant, then we only need to calculate the sum of internal edge-weights and the sum of external edge-weights.
- The time complexity of our method is $O(TlogT)$, where T is the number of non-zero edge-weights associated with all internal nodes. In our method, we first sort those edge-weights and then calculate related values by scanning this sorted list in a linear time. The first step costs $O(TlogT)$ and the second step requires $O(T)$.
- The time complexity for calculating the p-value of CCME is $O(n^2)$, where n is the number of vertices within the candidate community. Firstly, calculating the p-value for an internal node needs to visit all internal nodes, which spends $O(n)$. Then, for n internal nodes, $O(n^2)$ time is needed in total. Finally, sorting all p-values costs $O(nlogn)$. Thus, the total time complexity is $O(n^2)$.
- The time complexity for calculating the p-value in OSLOM is $O((N-n)^2)$, where N is the number of all vertices in the network and n is the number of vertices of the candidate community. The time is mainly composed of four parts: (1) Calculating a rank variable value for each vertex outside of the candidate community, which spends $O(d_{external})$. (2) Ranking the rank variable costs $O((N-n)log(N-n))$. (3) Based on the rank values, $O((N-n)^2)$ is needed to calculate the according cumulative distribution values. (4) Ranking the cumulative distribution values spends $O((N-n)log(N-n))$. Thus, the time complexity of the whole process is $O((N-n)^2)$.

External validation metrics

In our experiment, three external metrics are used to validate the identified communities based on the ground-truth information. Given the set of ground-truth communities C, two nodes are said to have the same label if they are contained in the same community from C. Then, each node pair with respect to the set of identified communities has four possibilities: (1) True Positive (TP): two nodes with the same label belong to the same community; (2) False Negative (FN): two nodes with the same label are distributed to different communities; (3) False Positive (FP): two nodes with different labels are allocated to the same community; (4) True Negative (TN): two nodes with different labels are distributed to different communities. Then, Jaccard, Precision and Recall are defined as follows:

$$Jaccard = \frac{TP}{TP+TN+FN},\ Precision = \frac{TP}{TP+FP},\ Recall = \frac{TP}{TP+FN}. \tag{8}$$

For each community ω_k in the set of identified communities Ω, we can find a ground-truth community c_b from C such that these two communities have the largest value of Jaccard or Precision or Recall. Then, the Jaccard, Precision and Recall for each identified community ω_k are defined as Equation 9, Equation 10 and Equation 11, respectively.

$$Jaccard(\omega_k, C) = max\left\{ \frac{|\omega_k \cap c_b|}{|\omega_k \cup c_b|} \middle| c_b \in C \right\}. \tag{9}$$

$$Precision(\omega_k, C) = max\left\{ \frac{|\omega_k \cap c_b|}{|\omega_k|} \middle| c_b \in C \right\}. \tag{10}$$

$$Recall(\omega_k, C) = max\left\{ \frac{|\omega_k \cap c_b|}{|c_b|} \middle| c_b \in C \right\}. \tag{11}$$

Materials

SLPAw is obtained from `https://sites.google.com/site/` and Infomap and Louvain are implemented in the R package igraph.[3]

Parameter setting

We use the default parameter setting of three community detection methods: Louvain, Inofmap and SLPAw. In particular, the parameter $-r$ of SLPAw is fixed to be 0.45 according to the suggestion in.[4]

Table 1: The detailed information of six un-weighted networks. N is the number of vertices, M is the number of edges, $\langle k \rangle$ is the average degree, and C_{max} (C_{min}) is the maximal (minimum) size of the ground-truth communities.

Data Set	N	M	$\langle k \rangle$	C_{max}	C_{min}
Karate	34	78	4.59	18	16
Football	115	613	10.57	13	5
Personal	561	8375	29.91	150	3
Polblogs	1490	19090	27.32	758	732
Polbooks	105	441	8.4	49	13
Railways	301	1226	6.36	46	1

Network information

Results

The Pearson's correlation coefficients between each internal metric and each external metric on weighted PPI networks and un-weighted networks are listed in Table 2, Table 3, Table 4 and Table 5, respectively.

In Table 2, Table 3 and Table 4, the set of protein complexes in CYC2008, MIPS and SGD is utilized as the set of ground-truth communities, respectively. The value of the left top corner is the name of ground-truth community set. The community detection methods and the external validation metrics used in this paper are listed in the leftmost column and the left second column, respectively. The internal validation metrics and the PPI networks are given in the topmost row and the second top row, respectively. Similarly, Table 5 presents the detailed results on un-weighted networks in which the columns and rows are arranged in the same manner. For convenience, "Modularity" and "Conductance" are abbreviated to "Modu." and "Cond.", respectively.

Table 2: The Pearson's correlation coefficient between each internal metric and each external metric on PPI networks when CYC2008 is utilized as the set of ground-truth communities.

CYC2008		Modu.	Cond.	OSLOM	Ours	CCME	Modu.	Cond.	OSLOM	Ours	CCME
		Collins2007					Gavin2006				
Louvain	Jaccard	-0.0677	0.0590	-0.0417	0.1480	0.0184	-0.1405	0.2878	0.1413	0.2994	0.0618
	Precision	-0.0728	0.0679	-0.0423	0.1368	0.0190	-0.1406	0.2964	0.1441	0.2995	0.0572
	Recall	0.1452	-0.1780	-0.0111	0.2838	-0.0475	0.3470	-0.4123	0.5697	0.3349	-0.0710
Infomap	Jaccard	-0.0125	0.2061	0.0173	0.1849	0.0061	0.1289	0.3482	0.2072	0.4364	0.0560
	Precision	-0.0220	0.1275	-0.0463	0.1647	0.0079	0.1214	0.2604	0.1933	0.4098	0.0620
	Recall	0.1195	0.0281	0.0362	0.2812	-0.0337	0.2344	0.0890	0.3990	0.2936	-0.0474
SLPAw	Jaccard	-0.0295	0.1331	0.0296	0.2068	0.0319	0.1029	0.2511	0.1941	0.4285	0.0586
	Precision	-0.0375	0.0510	-0.0464	0.1876	0.0350	0.0921	0.1439	0.2034	0.3947	0.0560
	Recall	0.1204	-0.0501	0.0514	0.2985	-0.0427	0.2470	0.0605	0.3559	0.2941	-0.0481
		Krogan2006_core					Krogan2006_extended				
Louvain	Jaccard	-0.1142	0.1374	0.1950	0.3727	0.0704	-0.2107	0.1833	0.0254	0.3349	0.0344
	Precision	-0.1701	0.2129	0.0821	0.3144	0.0794	-0.2560	0.2257	-0.0693	0.3471	0.0454
	Recall	0.4439	-0.6318	0.7467	0.2463	-0.0954	0.4482	-0.6952	0.8343	-0.1788	-0.0910
Infomap	Jaccard	0.0990	0.1886	0.3756	0.4089	0.0452	0.0805	0.2684	0.3834	0.3505	0.0473
	Precision	0.0685	0.2286	0.3355	0.3952	0.0503	0.0426	0.3064	0.3233	0.3712	0.0535
	Recall	0.2792	-0.1323	0.4493	0.1893	-0.0730	0.2888	0.0189	0.4934	0.0882	-0.0572
SLPAw	Jaccard	0.0114	0.1455	0.3900	0.4391	0.0550	-0.0237	0.2393	0.4427	0.3664	0.0702
	Precision	-0.0158	0.1671	0.3685	0.4206	0.0597	-0.0448	0.2420	0.4251	0.3638	0.0746
	Recall	0.2440	-0.1919	0.4603	0.1974	-0.0558	0.2548	0.0313	0.5007	0.1735	-0.0860

Table 3: The Pearson's correlation coefficient between each internal metric and each external metric on PPI networks when MIPS is utilized as the set of ground-truth communities.

MIPS		Modu.	Cond.	OSLOM	Ours	CCME	Modu.	Cond.	OSLOM	Ours	CCME
		Collins2007					Gavin2006				
Louvain	Jaccard	0.0921	0.0292	0.1528	0.4282	-0.0376	0.0566	0.1151	0.4166	0.5097	0.0237
	Precision	0.0537	0.0321	0.1121	0.3321	-0.0230	-0.0094	0.1790	0.2824	0.4234	0.0346
	Recall	0.2701	-0.1593	0.1498	0.5088	-0.0980	0.4732	-0.5119	0.7031	0.4748	-0.1077
Infomap	Jaccard	0.1174	0.1370	0.1201	0.4184	-0.0470	0.2492	0.3124	0.3325	0.5213	0.0188
	Precision	0.0748	0.0442	0.0111	0.3089	-0.0311	0.2096	0.2371	0.2420	0.4411	0.0280
	Recall	0.2257	0.0315	0.1521	0.4931	-0.0824	0.3404	0.1531	0.4527	0.4406	-0.0897
SLPAw	Jaccard	0.0860	0.0709	0.0724	0.4352	-0.0174	0.2011	0.2432	0.2700	0.5067	0.0270
	Precision	0.0515	0.0537	0.0156	0.3331	-0.0035	0.1535	0.1551	0.1567	0.4145	0.0333
	Recall	0.2138	-0.0067	0.1068	0.5149	-0.0921	0.3732	0.1534	0.3822	0.4859	-0.0917
		Krogan2006_core					Krogan2006_extended				
Louvain	Jaccard	0.0315	-0.0585	0.2648	0.3613	0.0487	-0.1782	0.1550	-0.0066	0.3245	0.0372
	Precision	-0.0698	0.0773	0.0700	0.2470	0.0613	-0.2594	0.2544	-0.1916	0.3423	0.0586
	Recall	0.5869	-0.7310	0.7442	0.2342	-0.1332	0.5229	-0.6341	0.7692	-0.1121	-0.1158
Infomap	Jaccard	0.1576	0.1553	0.3335	0.3334	0.0186	0.1327	0.2528	0.3774	0.3025	0.0241
	Precision	0.1008	0.1906	0.2657	0.2886	0.0317	0.0524	0.2555	0.2488	0.2862	0.0392
	Recall	0.3459	-0.0031	0.3828	0.2802	-0.1039	0.3672	0.1503	0.4809	0.2516	-0.0896
SLPAw	Jaccard	0.0519	0.0503	0.3155	0.2964	0.0345	0.0210	0.1965	0.3560	0.2776	0.0416
	Precision	0.0055	0.0690	0.2846	0.2619	0.0434	-0.0188	0.2058	0.3166	0.2604	0.0512
	Recall	0.3252	-0.1215	0.4011	0.2212	-0.1013	0.3703	0.1415	0.4948	0.2652	-0.1569

Table 4: The Pearson's correlation coefficient between each internal metric and each external metric on PPI networks when SGD is utilized as the set of ground-truth communities.

SGD		Modu.	Cond.	OSLOM	Ours	CCME	Modu.	Cond.	OSLOM	Ours	CCME
		Collins2007					Gavin2006				
Louvain	Jaccard	-0.0969	0.1099	0.0518	0.1179	0.0645	-0.1548	0.1578	0.2688	0.2044	0.0787
	Precision	-0.1068	0.1233	0.0521	0.0940	0.0678	-0.1594	0.1690	0.2664	0.2045	0.0814
	Recall	0.1605	-0.1271	0.0723	0.2496	-0.0635	0.4288	-0.5064	0.6481	0.3639	-0.0882
Infomap	Jaccard	-0.0703	0.1587	0.0144	0.1448	0.0617	0.0525	0.2668	0.2679	0.3197	0.0409
	Precision	-0.0756	0.1491	0.0202	0.1321	0.0649	0.0487	0.2499	0.2723	0.3149	0.0443
	Recall	0.0304	0.0148	0.0377	0.2270	0.0705	0.2221	0.0640	0.3967	0.2398	-0.0619
SLPAw	Jaccard	-0.0758	0.0539	0.0291	0.1665	0.0624	-0.0022	0.1811	0.2440	0.3249	0.0519
	Precision	-0.0815	-0.0202	-0.0381	0.1478	0.0665	-0.0060	0.0965	0.2395	0.3044	0.0551
	Recall	0.1280	-0.0894	0.0531	0.2771	-0.0573	0.2075	0.0023	0.3756	0.2413	-0.0609
		Krogan2006_core					Krogan2006_extended				
Louvain	Jaccard	-0.0937	0.0742	0.2553	0.3836	0.0636	-0.2244	0.1623	0.0690	0.3435	0.0496
	Precision	-0.1302	0.1351	0.1550	0.3234	0.0684	-0.2609	0.2276	-0.0473	0.3142	0.0538
	Recall	0.4786	-0.6800	0.7721	0.2318	-0.1041	0.4745	-0.7190	0.8646	-0.1828	-0.0981
Infomap	Jaccard	0.0878	0.1809	0.3909	0.3869	0.0303	0.0725	0.2738	0.3890	0.3279	0.0405
	Precision	0.0713	0.1995	0.3444	0.3649	0.0322	0.0322	0.2699	0.2998	0.3155	0.0467
	Recall	0.2637	-0.0557	0.4484	0.2175	-0.0917	0.2790	0.0764	0.4677	0.1164	-0.0792
SLPAw	Jaccard	0.0122	0.1213	0.4028	0.4162	0.0470	-0.0346	0.2339	0.4193	0.3357	0.0602
	Precision	-0.0061	0.1356	0.3503	0.3710	0.0507	-0.0547	0.2336	0.3694	0.3061	0.0657
	Recall	0.2780	-0.1418	0.4770	0.2461	-0.0691	0.2513	0.0650	0.5103	0.1722	-0.1055

Table 5: The Pearson's correlation coefficient between each internal metric and each external metric on un-weighted networks.

Pearson		Modu.	Cond.	OSLOM	Ours	CCME	Modu.	Cond.	OSLOM	Ours	CCME
		Karate					Football				
Louvain	Jaccard	0.9952	0.5755	-0.0028	-0.2635	-0.9147	-0.2472	0.4831	0.1646	0.7112	0.1437
	Precision	-0.7135	-0.4762	0.5725	-0.0107	0.4606	-0.4465	0.3801	0.2882	0.7620	0.2257
	Recall	0.9998	0.5849	-0.0632	-0.2436	-0.8983	0.4704	0.2393	-0.2985	-0.1363	-0.1966
Infomap	Jaccard	0.9892	0.9328	0.8486	-0.9809	-0.9161	0.8204	0.8914	0.6823	0.8691	0.7001
	Precision	-0.1799	0.3286	0.5000	0.2277	0.4317	0.7630	0.8660	0.6670	0.8701	0.6937
	Recall	0.9973	0.9038	0.8074	-0.9925	-0.9431	0.8451	0.9008	0.7364	0.8483	0.7388
SLPAw	Jaccard	0.9981	0.9023	0.0729	-0.9595	-0.9217	-0.3860	0.3086	-0.0035	0.7251	0.4347
	Precision	Nan	Nan	Nan	Nan	Nan	-0.6551	0.0468	0.1469	0.6501	0.5568
	Recall	0.9981	0.9023	0.0729	-0.9595	-0.9217	0.5554	0.8206	-0.3652	0.5226	-0.2206
		Personal					PolBlogs				
Louvain	Jaccard	0.6372	-0.2661	0.6552	0.6904	-0.3310	0.6129	0.6368	0.9733	0.1291	0.1437
	Precision	0.3104	0.3625	-0.2318	0.3172	-0.0420	0.6284	-0.4391	0.2877	0.6566	0.2257
	Recall	0.5687	-0.3208	0.7353	0.6214	-0.3589	0.6129	0.6363	0.9712	0.1295	-0.1966
Infomap	Jaccard	0.6614	0.3852	0.4000	0.7670	-0.3608	0.9997	0.5824	0.4831	0.9328	-0.0673
	Precision	0.2645	0.4536	-0.2139	0.3482	-0.1110	0.0354	0.1291	-0.1822	0.0666	0.0304
	Recall	0.6098	0.3489	0.4616	0.7059	-0.3561	0.9995	0.5818	0.4820	0.9285	-0.0680
SLPAw	Jaccard	0.6409	0.3816	0.3910	0.7471	-0.4174	0.9957	-0.2670	0.5755	0.9801	-0.5727
	Precision	0.1419	0.0619	-0.3568	0.1821	-0.1030	-0.9492	0.2420	-0.5275	-0.8185	0.1960
	Recall	0.6237	0.4069	0.4204	0.7190	-0.4024	0.9975	-0.2668	0.5754	0.9756	-0.5546
		PolBooks					Railway				
Louvain	Jaccard	0.9986	0.9351	-0.4555	0.8765	0.2806	0.2793	0.0711	0.5508	0.7033	0.0448
	Precision	0.8944	0.6481	-0.1508	0.5249	-0.1215	-0.7761	0.5527	-0.6410	-0.6742	0.3636
	Recall	0.9945	0.9122	-0.3650	0.8638	0.2020	0.7903	-0.1685	0.5701	0.8998	-0.0110
Infomap	Jaccard	0.9934	0.9234	0.3044	0.8082	-0.9091	0.4665	0.3796	0.5333	0.7161	0.0633
	Precision	0.3060	-0.0307	-0.4003	0.0856	-0.3127	-0.5271	0.0686	-0.3528	-0.4191	-0.1775
	Recall	0.9746	0.9549	0.3698	0.8167	-0.8873	0.5073	0.2090	0.5506	0.7135	0.1123
SLPAw	Jaccard	0.9923	0.9379	-0.9905	0.9613	-0.5477	0.2733	0.1389	0.4448	0.5868	0.1707
	Precision	0.8493	0.5191	-0.7822	0.6227	-0.4181	-0.7016	0.0821	-0.4803	-0.6105	-0.2174
	Recall	0.9704	0.9730	-0.9807	0.9810	-0.5523	0.3908	0.0615	0.4288	0.6463	0.1805

References

[1] Mark E. N., Michelle G., (2004). Finding and evaluating community structure in networks. *Physical Review E*, 69(2): 026113.

[2] Jure L., Kevin J L., Anirban D., Michael W M., (2009). Community structure in large networks: Natural cluster sizes and the absence of large well-defined clusters. *Internet Mathematics*, 6(1):29–123.

[3] Gabor C., Tamas N., (2006). The igraph software package for complex network research. *InterJournal, complex systems*, 1695(5):1–9.

[4] Jierui X., Boleslaw K S., Xiaoming L., (2011). Slpa: Uncovering overlapping communities in social networks via a speaker-listener interaction dynamic process. In *IEEE 11th international conference on data mining workshops*, pages 344–349.
